# Prevalence of dental caries and associated factors among school-aged children in Tripoli, Libya: a cross-sectional study

**DOI:** 10.1186/s12903-021-01545-9

**Published:** 2021-04-30

**Authors:** Hosam Alraqiq, Ahmid Eddali, Reema Boufis

**Affiliations:** 1 Section of Growth and Development, Division of Pediatric Dentistry, College of Dental Medicine, Irving Medical Center, Columbia University, 622 W 168th St, New York, NY 10032 USA; 2 School Health Program, Tripoli, Libya; 3 College of Dental Medicine, Zawia University, Zawia, Libya

**Keywords:** Dental caries, Children, Oral health, Prevalence

## Abstract

**Background:**

In many developing countries, the prevalence of dental caries has increased due to lifestyle changes, lack of preventive services, and inadequate access to dental care. In Arab countries, the increased prevalence of caries has correlated with economic growth over the past decades, resulting in greater access to unhealthy foods and higher consumption of sugar, particularly among children. However, few studies have assessed caries prevalence among pediatric populations in Arab countries. The objective of this study was to assess the prevalence of dental caries and factors associated with caries among children in Tripoli, Libya.

**Methods:**

This cross-sectional study included a convenience sample of 1934 children in first grade (age 6–7 years, n = 1000) and seventh grade (age 11–12 years, n = 934). Four health centers in Tripoli were selected for screening based on location and participation in school-entry health examinations. Data were collected through self-administered parent surveys and visual dental screenings by trained examiners from September 24 to October 15, 2019. The survey comprised questions about socioeconomic characteristics and oral health behaviors, including toothbrushing, sugar consumption, and dental care history. During screenings, untreated decay, missing teeth, and filled teeth (DMFT or dmft) were recorded. Prevalence of tooth decay was calculated as the proportion of children with high DMFT/dmft scores. Binary logistic and negative binomial regression analyses (with significance at *p* ≤ 0.05) were used to assess factors associated with caries.

**Results:**

Among 1000 first-grade children, 78.0% had decay in their primary teeth, with a mean dmft of 3.7. Among 934 seventh-grade children, 48.2% had caries in their permanent teeth, with a mean DMFT of 1.7. The most significant factors associated with caries prevalence were socioeconomic, such as screening site (first grade, *p* = 0.02; seventh grade, *p* < 0.001) and maternal employment (seventh grade, *p* = 0.02), and behavioral, such as toothbrushing duration (seventh grade, *p* = 0.01), past dental treatment (both grades, *p* < 0.001), and past emergency visit (both grades, *p* < 0.001).

**Conclusions:**

Caries prevalence was associated with several behavioral and socioeconomic factors, including screening site, maternal employment, toothbrushing duration, past dental treatment, and past emergency visit. Efforts should be made to address these factors to minimize barriers and improve oral health behavior and care utilization. These findings can be used to evaluate current public health initiatives and inform future planning.

**Supplementary Information:**

The online version contains supplementary material available at 10.1186/s12903-021-01545-9.

## Background

Dental caries is the most common noncommunicable childhood disease, resulting in mineral loss of hard tissue. The development of caries lesions is associated with many factors, including biological, behavioral, and socioeconomic determinants [1, 2]. Some of the primary factors underlying dental caries include poor dietary habits and oral hygiene behaviors, *Streptococcus mutans* infection, anomalies of tooth development, and lack of dental care [3]. Left untreated, caries can lead to pain, infection, lost school days, problems with speech and language development, and other long-term effects that adversely impact quality of life [4–6]. In addition, dental caries is associated with socioeconomic disparities, as it disproportionally affects low-income and racially or ethnically diverse populations [1]. Thus, individual and population-based initiatives to promote oral health among all socioeconomic groups have become common worldwide [7].

In many developing countries, the prevalence of caries has been steadily increasing, largely due to lifestyle changes, the lack of oral health preventive services, and inadequate access to dental care [8]. In Arab countries, the increased prevalence of caries has been associated with the economic growth that has occurred over the past several decades, which has resulted in greater access to unhealthy foods and increased consumption of sugar [3]. A lack of awareness about oral health practices has also contributed to the increase in caries [9]. Despite these issues, few studies have been conducted to assess the prevalence of caries in developing Arab countries, particularly in Libya, for which data regarding the oral health status of citizens are scarce [10]. In 2019, when this study was conducted, the population of Tripoli was approximately 1.16 million, and the estimated proportion of Libyan children between the ages of 0 and 14 years was 25% (exact data are lacking, as Libya does not conduct a census or have official demographic statistics available due to political instability) [11]. Despite the large proportion of children in Tripoli, the only study to examine dental caries among a pediatric population dates back to 1991 [12].

All children entering first and seventh grade in Tripoli are required to receive a school-entry health examination that includes oral health screening. Each of Tripoli’s 5 districts designates several health centers to administer this examination annually. However, due to lack of resources and competing priorities, data from the screening program have not been consistently analyzed.

The results of this study will assist in the evaluation of current public health initiatives as well as program and policy planning in Tripoli. The findings may also provide novel insights regarding the socioeconomic and behavioral factors associated with pediatric dental caries among populations in developing countries, which can be used to inform oral health planning, formulate education-based preventive strategies to positively impact oral health–related behavior and utilization, and target specific populations with a high prevalence of dental caries.

The study’s objective was to collect current data to determine the prevalence of dental caries and its associated factors among school-aged children in Tripoli. Specific aims were to (1) determine the prevalence of dental caries among first- and seventh-grade children in Tripoli and (2) examine the socioeconomic and behavioral factors associated with dental caries prevalence.

## Methods

### Sampling

This cross-sectional study was designed as part of a Fulbright Alumni Action grant sponsored by the US Department of State and administered by America-Mideast Educational and Training Services (AMIDEAST). The survey was implemented in coordination with the Tripoli School Health Administration’s Bureau of Primary Care. The study protocol was approved by the Tripoli Bureau of Primary Care (School Health–Tripoli protocol number P121) and the institutional review board of the Columbia University Irving Medical Center (protocol number AAAT0022). An informative letter and consent form, written in Arabic, was presented to all participants, and written informed consent was obtained from all parents at the time of their child’s dental screening (and before screening was performed). In addition to granting consent for their child’s participation, parents were invited to complete a survey (Additional file 1) at the time of screening; this survey included questions about basic socioeconomic characteristics and items regarding their child’s core oral health behaviors, including toothbrushing habits, sugar consumption, and dental visit history. The survey was self-administered, and a trained examiner was available to answer questions or provide clarification as needed.

The study used primary data collected from a convenience sample of 1934 participants comprising first-grade (aged 6–7 years, n = 1000) and seventh-grade (aged 11–12 years, n = 934) children in Tripoli during their 2019 school-entry health examinations. No inclusion criteria (with the exception of school grade) or exclusion criteria were applied. The specific school grades were selected based on the typical timing of dentition and the lower likelihood of encountering mixed dentition during the examinations (i.e., children in first grade have mostly primary teeth, with few permanent teeth present, and children in seventh grade have mostly permanent teeth, with few primary teeth present).

Because the exact number of children in Tripoli was not available due to the lack of official statistics, random (probability) sampling was not possible [13]. Therefore, we used convenience (nonprobability) sampling, and we oversampled to decrease the potential for sampling errors and increase the validity of the study’s results [13]. All children were screened on a first-come, first-served basis until either a maximum number of 1000 children per group was achieved (which occurred for the first-grade group) or the school examination campaign ended (which occurred for the seventh-grade group). Using this approach, we estimated that we engaged approximately 10% of the first-grade and seventh-grade children in Tripoli, indicating a representative sample size based on the sampling formula developed by Cochran [14].

A convenience sample of first- and seventh-grade children was screened during the same time as, but independent from, their 2019 school-entry examinations in 2 socioeconomically diverse districts. Four community health centers were selected based on their geographic location and their participation in school-entry examinations. Two centers were located in the relatively low-income district of Abu Saleem, and 2 were located in the relatively high-income district of Hey al Andalus. A total of 500 first- and seventh-grade children were screened at each health center, with the exception of 1 center, in which only 434 children in seventh grade were screened.

### Data collection

Among the demographic factors included in the parent survey were children’s sex and school type (public or private) and parents’ self-reported educational level (less than high school or high school and higher) and employment status (employed, freelancer, or homemaker). The employed category included parents with any job at a company, organization, or government institution; the freelance category included parents with any business-related self-employment; and the homemaker category included mothers who were not employed outside the home.

Information collected on children’s sugar intake included the consumption of soda, juice, and sweets (ranging from rarely or never to ≥ 4 times per day). Toothbrushing habits included brushing frequency (ranging from rarely or never to ≥ 4 times per day) and amount of time spent per brushing (ranging from < 1 min to ≥ 3 min, with parents controlling and monitoring the time). Care utilization included time since past dental visit (never, within the past year, or more than 1 year), main reason for past dental visit (routine check-up, treatment, or emergency), and main barrier to care utilization (financial considerations, lack of time, lack of knowledge regarding where to go, lack of trust in dentists, or other barriers specified by parents) for children who had never visited the dentist (Additional file 1).

To ensure objective criteria were consistently used during the dental screenings of children, half-day (i.e., 4–5 h) calibration training was provided on September 21, 2019, to the 14 dentists who served as screening examiners (κ values were not calculated). Our training process was guided by the basic screening protocol of the American State and Territorial Dental Directors (ASTDD), [15] which recommends that the initial session comprise 2–3 h of didactic training. Based on these guidelines and the fact that all participating dentists had been practicing dentistry for a minimum of 5 years, a half-day session was deemed appropriate. The training included an in-person presentation, 2 training videos, a test that was administered toward the end of the session, and a post-test discussion. In accordance with ASTDD recommendations, [15] after the initial half-day training session, all examiners participated in a 2- to 3-h clinical practice session with the directors of their respective health centers. During the practice session, the examiners applied the skills they had learned in training and discussed potential differences in the interpretation of screening criteria under real-world clinical conditions.

Daily visual dental screenings were performed from September 24 to October 15, 2019. The screening process was based on ASTDD protocol [15]. All examiners used a reference guide that included photographs and instructions regarding how to evaluate and score teeth for dental caries. Screenings were conducted at the 4 participating community health centers during school-entry examinations and were performed via visual assessment only, using disposable dental mirrors, tongue depressors, and flashlights. Dental explorers were not used; however, gauze and toothpicks were used to clean teeth and remove food if necessary.

Two examiners worked together during the screening process. The first examiner performed the visual dental examination, during which he or she verbally reported all findings to the second examiner, who recorded the data. Data collected included the presence or absence of teeth, the number of teeth with cavitated lesions and fillings, and the number of missing teeth due to decay. Mixed dentition status was not recorded. For first-grade children, we were interested in primary teeth only, as few permanent teeth are present at age 6–7 years; for seventh-grade children, we were interested in permanent teeth only, as few primary teeth remain at age 11–12 years and most teeth are permanent. After the screening, all children received free dental products and flyers about the importance of oral health.

### Statistical analysis

The primary outcome was the prevalence of untreated and treated dental caries (i.e., caries experience), which was determined by calculating the proportion of children with any decayed, missing, or filled tooth (uppercase DMFT indicates permanent teeth, and lowercase dmft indicates primary teeth) due to dental decay. The severity of tooth decay was also measured by calculating the mean DMFT or dmft score and the Significant Caries (SiC) Index score, as recommended by the World Health Organization [16].

Binary logistic regression analysis was used to examine the association between the independent variables and the prevalence of untreated caries, and negative binomial regression analysis was used to assess factors associated with caries severity (DMFT and dmft). All socioeconomic and behavioral variables were included in the multivariable models (vs inclusion of only the variables that were statistically significant in the bivariate analysis) based on their association with dental caries, as reported in the literature. This approach was also used to minimize the risk of systemic biases in the statistical results (e.g., odds ratios [ORs], *p* values, and 95% confidence intervals [CIs]) that can occur due to the application of variable selection [17]. All multivariable models were evaluated for significance using the goodness of fit method.

Statistical significance was set at *p* ≤ 0.05. For categories, overall *p* values were obtained to assess whether a variable with more than 2 levels met the screening threshold. For variables, *p* values were calculated to determine between-level significance compared with the reference category. Data analyses were performed using IBM SPSS Statistics software, version 25.0 (IBM Corp.).

Unemployed fathers and deceased parents (both mothers and fathers) were categorized as missing data in the bivariate analysis because the majority of parents surveyed were employed, freelancers, or homemakers (i.e., among 1000 first-grade children, there were only 14 deceased parents [1.4%, 2 mothers and 12 fathers] and 18 unemployed parents [1.8%, 0 mothers and 18 fathers]). Given the small proportion of parents who were unemployed or deceased, these categories were not a good fit for any of the dichotomized variables (e.g., employed/homemaker or employed/freelancer).

## Results

Among the 1000 first-grade children screened, the proportion of boys and girls was similar (50.1% vs. 49.9%, respectively), and most of the children were enrolled in public schools (73.6%) compared with private schools (25.2%). Most parents of first-grade children had a high school or greater educational level, with more mothers (75.4%) completing high school than fathers (68.4%). With regard to employment status, 53.9% of mothers were homemakers and 46.1% were employed; 66.7% of fathers were employed and 33.3% were freelancers. Similar demographic patterns were observed for the 934 seventh-grade children (Table 1).

**Table 1:** Socioeconomic and demographic characteristics of participating children by school grade

Characteristic	First grade, N (%) (n = 1000)		Seventh grade, N (%) (n = 934)	
	Total participants	Missing data	Total participants	Missing data
Sex				
Male	492 (50.1)	18 (1.8)	419 (44.9)	1 (0.1)
Female	490 (49.9)		514 (55.1)	
School type				
Public	703 (73.6)	45 (4.5)	823 (88.1)	0
Private	252 (25.2)		111 (11.9)	
Screening site				
Abu Saleem	500 (50.0)	0	430 (46.0)	0
Hey al Andulus	500 (50.0)		504 (54.0)	
Mother’s educational level				
< High school	221 (24.6)	100 (10.0)	277 (29.8)	4 (0.4)
≥ High school	679 (75.4)		653 (70.2)	
Father’s educational level				
< High school	280 (31.6)	113 (11.3)	318 (34.3)	7 (0.7)
≥ High school	607 (68.4)		609 (65.7)	
Mother’s employment status				
Homemaker	424 (53.9)	214 (21.4)	448	34 (3.6)
Employed	362 (46.1)		452 (50.2)	
Father’s employment status				
Freelancer	273 (33.3)	181 (18.1)	315 (33.7)	26 (2.7)
Employed	546 (66.7)		593 (65.3)	

Among first-grade children, 78.0% had dental decay in their primary teeth, with a mean dmft of 3.7 ± 3.3 and a mean SiC score of 7.45 ± 2.34. Most of the children with caries had untreated decay (76.9%), and small percentages had fillings (6.3%) or missing teeth (5.3%) due to decay. Among seventh-grade children, 48.2% had dental decay in their permanent teeth, with a mean DMFT of 1.7 ± 1.6 and a mean SiC score of 3.06 ± 3.58. Most of the children with caries had untreated decay (43.6%), and small percentages had fillings (10.0%) or missing teeth (6.7%) due to decay (Table 2).

**Table 2:** Dental caries among first- and seventh-grade children in Tripoli, Libya

School grade	N (%)				Severity score, mean ± SD^c^	SiC score, mean ± SD^d^
	Decayed^a^	Missing	Filled	Total decay^b^		
*First grade (n* = *1000)*					dmft	dmft
Total	766 (76.9)	53 (5.3)	63 (6.3)	777 (78.0)	3.7 ± 3.3	7.5 ± 2.3
Abu Saleem	382 (77.0)	19 (3.8)	37 (7.5)	386 (77.8)	3.7 ± 3.3	7.4 ± 2.3
Hey al Andalus	384 (76.8)	34 (6.8)	26 (2.5)	391 (78.2)	3.7 ± 3.3	7.8 ± 2.4
*Seventh grade (n* = *934)*					DMFT	DMFT
Total	407 (43.6)	63 (6.7)	59 (10.0)	450 (48.2)	1.7 ± 1.6	3.1 ± 3.6
Abu Saleem	135 (45.3)	28 (6.5)	59 (13.7)	210 (48.8)	1.4 ± 1.9	3.1 ± 1.8
Hey al Andalus	212 (42.1)	35 (6.9)	35 (6.9)	240 (47.6)	1.0 ± 1.4	3.0 ± 1.3

*SiC* significant caries index, *DMFT* delayed, missing, or filled permanent teeth, *dmft* delayed, missing, or filled primary teeth

^a^Decayed tooth status includes untreated lesions or decay

^b^Total decay (or caries experience) includes any child with any tooth that is decayed, missing, or filled due to dental decay

^c^Severity score is the mean number of decayed, missing, or filled teeth (DMFT or dmft), both treated and untreated

^d^SiC score is the mean DMFT or dmft of one-third of the study group with the highest caries score

Parents were also asked about the main barrier preventing them from taking their children to the dentist. Barriers for second-grade children, from most to least frequently cited, included lack of need to see the dentist (76.7%, n = 549), lack of time (13.3%, n = 95), financial reasons (3.5%, n = 25), uncertainty about where to go for dental services (2.8%, n = 20), fear of the dentist (2.2%, n = 16), and other reasons (1.5%, n = 11). Similar patterns were observed for first-grade children (Table 2).

### Socioeconomic and behavioral factors associated with dental caries

With regard to caries prevalence, among first-grade children, the most significant factors associated with untreated decay were screening site (adjusted OR [aOR] = 0.51, 95% CI = 0.29–0.90, *p* = 0.02), past dental treatment (aOR = 3.81, 95% CI = 1.82–7.95, *p* < 0.001), and past emergency visit (aOR = 9.91, 95% CI = 3.47–28.30, *p* < 0.001). Among seventh-grade children, the most significant factors associated with untreated decay were screening site (aOR = 0.48, 95% CI = 0.32–0.73, *p* < 0.001), maternal employment (aOR = 0.62; 95% CI = 0.43–0.88, *p* = 0.02), brushing duration of 3 min or more (aOR = 0.39, 95% CI = 0.19–0.79, *p* = 0.01), past dental treatment (aOR = 2.23, 95% CI = 1.49–3.33, *p* < 0.001), and past emergency visit (aOR = 2.76, 95% CI = 1.75–4.34, *p* < 0.001) (Table 3).

**Table 3:** Prevalence of untreated decay by socioeconomic characteristic, sugar consumption, toothbrushing behavior, and dental visit history

Child characteristic	First grade				Seventh grade			
	Unadjusted		Adjusted		Unadjusted		Adjusted	
	OR (95% CI)	*p* value	OR (95% CI)	*p* value	OR (95% CI)	*p* value	OR (95% CI)	*p* value
Socioeconomic characteristics								
Sex								
Male	1.0 [Ref]		1.0 [Ref]		1.0 [Ref]		1.0 [Ref]	
Female	0.81 (0.60–1.09)	0.16	0.77 (0.50–1.19)	0.24	1.12 (0.86–1.45)	0.40	1.39 (0.99–1.96)	0.06
School type								
Public	1.0 [Ref]		1.0 [Ref]		1.0 [Ref]		1.0 [Ref]	
Private	0.95 (0.68–1.33)	0.76	1.18 (0.70–1.97)	0.53	0.80 (0.53–1.20)	0.27	0.68 (0.39–1.17)	0.16
Screening site								
Abu Saleem								
Hey al Andalus	0.98 (0.73–1.32)	0.94	0.51 (0.29–0.90)	0.02	0.86 (0.67–1.13)	0.31	0.48 (0.32–0.73)	< 0.001
Mother’s educational level								
< High school	1.0 [Ref]		1.0 [Ref]		1.0 [Ref]		1.0 [Ref]	
≥ High school	0.79 (0.55–1.15)	0.22	0.91 (0.51–1.63)	0.75	0.88 (0.66–1.17)	0.38	1.13 (0.74–1.72)	0.56
Father’s educational level								
< High school	1.0 [Ref]		1.0 [Ref]		1.0 [Ref]		1.0 [Ref]	
≥ High school	0.76 (0.55–1.07)	0.12	0.83 (0.49–1.42)	0.51	1.05 (0.80–1.38)	0.72	1.07 (0.71–1.60)	0.75
Mother’s employment status								
Homemaker	1.0 [Ref]		1.0 [Ref]		1.0 [Ref]		1.0 [Ref]	
Employed	1.08 (0.78–1.50)	0.64	0.95 (0.61–1.47)	0.82	0.72 (0.56–0.94)	0.02	0.62 (0.43–0.88)	0.02
Father’s employment status								
Freelancer	1.0 [Ref]		1.0 [Ref]		1.0 [Ref]		1.0 [Ref]	
Employed	0.93 (0.66–1.31)	0.67	0.89 (0.55–1.43)	0.62	1.31 (0.99–1.72)	0.06	1.32 (0.92–1.89)	0.13
Sugar consumption								
Drinking soda		< 0.001		0.42		0.86		0.32
Rarely or never	1.0 [Ref]		1.0 [Ref]		1.0 [Ref]		1.0 [Ref]	
Several times/wk	1.79 (1.27–2.53)	< 0.001	1.61 (0.98–2.67)	0.33	0.97 (0.70–1.35)	0.87	0.71 (0.47–1.08)	0.11
1 time/d	1.93 (1.19–3.15)	0.01	1.43 (0.68–3.02)	0.55	1.26 (0.81–1.94)	0.30	0.80 (0.44–1.44)	0.46
2–3 times/d	1.97 (1.06–3.66)	0.03	1.87 (0.73–4.79)	0.50	1.64 (1.06–2.54)	0.03	1.11 (0.59–2.11)	0.74
≥ 4 times/d	2.26 (0.65–7.92)	0.20	3.45 (0.28–42.5)	0.64	1.71 (0.51–5.75)	0.39	1.88 (0.26–13.3)	0.53
Drinking juice		< 0.001		0.22		0.37		0.56
Rarely or never	1.0 [Ref]		1.0 [Ref]		1.0 [Ref]		1.0 [Ref]	
Several times/wk	2.78 (1.79–4.34)	< 0.001	2.01 (0.97–4.14)	0.32	1.17 (0.74–1.85)	0.50	1.46 (0.79–2.70)	0.22
1 time/d	2.76 (1.70–4.48)	< 0.001	1.52 (0.68–3.40)	0.95	1.34 (0.82–2.18)	0.24	1.54 (0.79–2.99)	0.20
2–3 times/d	3.34 (2.01–5.53)	< 0.001	1.81 (0.75–4.36)	0.64	1.53 (0.93–2.52)	0.09	1.87 (0.90–3.89)	0.10
≥ 4 times/d	2.80 (1.23–6.38)	0.01	2.09 (0.49–9.00)	0.82	1.65 (0.65–4.18)	0.30	1.31 (0.40–4.25)	0.65
Eating sweets		< 0.001		0.03		0.04		0.33
Rarely or never	1.0 [Ref]		1.0 [Ref]		1.0 [Ref]		1.0 [Ref]	
Several times/wk	2.29 (1.38–3.79)	< 0.001	1.83 (0.76–4.41)	0.88	0.99 (0.62–1.58)	0.96	0.77 (0.41–1.46)	0.42
1 time/d	2.60 (1.52–4.43)	< 0.001	2.71 (1.05–6.95)	0.33	0.91 (0.54–1.53)	0.72	0.68 (0.33–1.39)	0.29
2–3 times/d	3.70 (2.08–6.58)	< 0.001	3.49 (1.25–9.70)	0.09	1.29 (0.76–2.19)	0.35	0.85 (0.39–1.84)	0.67
≥ 4 times/d	2.01 (0.94–4.26)	0.07	1.11 (0.31–3.91)	0.03	2.22 (1.10–4.49)	0.03	1.58 (0.59–4.23)	0.37
Toothbrushing behavior								
Brushing frequency		0.01		0.91		0.37		0.59
Rarely or never	1.0 [Ref]		1.0 [Ref]		1.0 [Ref]		1.0 [Ref]	
Several times/wk	1.31 (0.80–2.16)	0.29	1.57 (0.40–6.19)	0.43	1.12 (0.71–1.75)	0.64	NA^a^	NA^a^
1 time/d	0.94 (0.59–1.50)	0.79	1.36 (0.35–5.25)	0.74	0.80 (0.51–1.23)	0.31	0.76 (0.49–1.16)	0.20
2–3 times/d	0.90 (0.56–1.45)	0.67	1.58 (0.40–6.20)	0.52	0.92 (0.59–1.44)	0.72	0.87 (0.55–1.36)	0.53
≥ 4 times/d	0.39 (0.21–0.75)	0.01	1.87 (0.39–8.95)	0.72	0.76 (0.41–1.40)	0.37	0.71 (0.36–1.43)	0.34
Brushing duration		< 0.001		0.02		< 0.001		< 0.001
< 1 min	1.0 [Ref]		1.0 [Ref]		1.0 [Ref]		1.0 [Ref]	
1 min	0.64 (0.40–1.03)	0.07	1.53 (0.83–2.80)	0.10	1.28 (0.85–1.92)	0.23	1.34 (0.83–2.15)	0.23
2 min	0.46 (0.28–0.76)	< 0.001	0.96 (0.51–1.81)	0.90	0.81 (0.53–1.24)	0.32	0.76 (0.47–1.25)	0.29
≥ 3 min	0.42 (0.24–0.72)	< 0.001	0.53 (0.25–1.13)	0.10	0.53 (0.29–0.96)	0.04	0.39 (0.19–0.79)	0.01
Dental visit history								
Past dental visit		< 0.001		< 0.001		< 0.001		< 0.001
Never	1.0 [Ref]		1.0 [Ref]		1.0 [Ref]		1.0 [Ref]	
Routine	1.08 (0.71–1.64)	0.73	0.78 (0.39–1.55)	0.48	1.80 (1.15–2.70)	< 0.001	1.71 (0.92–3.16)	0.09
Treatment	2.74 (1.60–4.70)	< 0.001	3.81 (1.82–7.95)	< 0.001	1.94 (1.41–2.67)	< 0.001	2.23 (1.49–3.33)	< 0.001
Emergency	14.1 (5.15–38.80)	< 0.001	9.91 (3.47–28.30)	< 0.001	2.35 (1.61–3.43)	< 0.001	2.76 (1.75–4.34)	< 0.001

*CI* confidence interval, *NA* not applicable, *OR* odds ratio

With regard to caries severity, among first-grade children, the most significant factors associated with caries severity were eating sweets 2–3 times per day (adjusted rate ratio [aRR] = 1.79, 95% CI = 1.06–3.03, *p* = 0.03), past dental treatment (aRR = 1.91, 95% CI = 1.44–2.52, *p* < 0.001), and past emergency visit (aRR = 2.01, 95% CI = 1.55–2.61, *p* < 0.001). Among seventh-grade children, the most significant factors associated with caries severity were screening site (aRR = 0.59, 95% CI = 0.45–0.79, *p* < 0.001), maternal employment (aRR = 0.73, 95% CI = 0.57–0.93, *p* = 0.02), brushing duration of 1 min (aRR = 1.47, 95% CI = 1.05–2.05, *p* = 0.03), past dental treatment (aRR = 2.61, 95% CI = 1.91–3.57, *p* < 0.001), and past emergency visit (aRR = 2.25, 95% CI = 1.70–2.99, *p* < 0.001) (Table 4).

**Table 4:** Severity of dental caries by socioeconomic characteristic, sugar consumption, toothbrushing behavior, and dental visit history

Child characteristics		First grade								Seventh grade						
		Unadjusted				Adjusted				Unadjusted				Adjusted		
		RR (95% CI)		*p* value		RR (95% CI)		*p* value		RR (95% CI)		*p* value		RR (95% CI)		*p* value
Socioeconomic characteristics																
Sex																
Male		1.0 [Ref]				1.0 [Ref]				1.0 [Ref]				1.0 [Ref]		
Female		0.93 (0.81–1.07)		0.32		0.94 (0.77–1.14)		0.52		1.00 (0.85–1.21)		0.88		1.16 (0.92–1.46)		0.22
School type																
Public		1.0 [Ref]				1.0 [Ref]				1.0 [Ref]				1.0 [Ref]		
Private		0.92 (0.78– 1.08)		0.32		1.00 (0.79–1.27)		0.18		1.02 (0.781.34)		0.88		0.78 (0.54–1.13)		0.18
Screening site																
Abu Saleem		1.0 [Ref]				1.0 [Ref]				1.0 [Ref]				1.0 [Ref]		
Hey al Andalus		1.00 (0.86–1.14)		0.95		0.51 (0.29–0.90)		0.23		0.73 (0.62–0.87)		< 0.001		0.59 (0.45–0.79)		< 0.001
Mother’s educational level																
< High school		1.0 [Ref]				1.0 [Ref]				1.0 [Ref]				1.0 [Ref]		
≥ High school		0.86 (0.73–1.03)		1.00		0.84 (0.65–1.08)		0.16		1.07 (0.88–1.29		0.51		1.05 (0.78–1.40)		0.76
Father’s educational level																
< High school		1.0 [Ref]				1.0 [Ref]				1.0 [Ref]				1.0 [Ref]		
≥ High school		0.91 (0.78–1.07)		0.12		0.97 (0.77–1.22)		0.77		1.20 (0.99–1.45)		0.06		1.28 (0.96–1.71)		0.10
Mother’s employment status																
Homemaker		1.0 [Ref]				1.0 [Ref]				1.0 [Ref]				1.0 [Ref]		
Employed		0.99 (0.84–1.16)		0.84		1.00 (0.82–1.23)		0.97		0.89 (0.74–1.06		0.19		0.73(0.57–0.93)		0.02
Father’s employment status																
Freelancer		1.0 [Ref]				1.0 [Ref]				1.0 [Ref]				1.0 [Ref]		
Employed		0.98 (0.83–1.16)		0.84		0.98 (0.79–1.22)		0.84		1.30 (1.08–1.58)		0.01		1.25 (0.97–1.61)		0.08
Sugar consumption																
Drinking soda				< 0.001				0.57				0.12				0.32
Rarely or never		1.0 [Ref]				1.0 [Ref]				1.0 [Ref]				1.0 [Ref]		
Several times/wk		1.44 (1.22–1.70)		< 0.001		1.50 (0.58–3.91)		0.41		0.78 (0.63–0.98		0.04		0.74 (0.55–0.98)		0.04
1 time/d		1.25 (1.00–1.56)		0.05		1.03 (0.69–1.54)		0.88		0.99 (0.74–1.31		0.92		0.76 (0.50–1.15)		0.19
2–3 times/d		1.37 (1.04–1.80)		0.03		1.05 (0.75–1.46)		0.80		1.02 (0.77–1.37		0.88		0.83 (0.54–1.28)		0.40
≥ 4 times/d		1.67 (1.00–2.77)		0.05		1.19 (0.95–1.50)		0.13		1.08 (0.49–2.39)		0.85		1.13 (0.32–4.04)		0.85
Drinking juice				< 0.001				0.22				0.87				0.52
Rarely or never		1.0 [Ref]				1.0 [Ref]				1.0 [Ref]				1.0 [Ref]		
Several times/wk		1.97 (1.53–2.53)		< 0.001		1.42 (0.97–2.08)		0.14		0.95 (0.71–1.29)		0.77		0.97 (0.43–2.18)		0.93
1 time/d		1.89 (1.44–2.47)		< 0.001		1.26 (0.83–1.90)		0.28		0.91 (0.66–1.26)		0.58		1.44 (0.89–2.35)		0.14
2–3 times/d		2.09 (1.60–2.74)		< 0.001		1.39 (0.90–2.16)		0.07		1.02 (0.73–1.42		0.91		1.25 (0.80–1.94)		0.33
≥ 4 times/d		2.58 (1.73–3.84)		< 0.001		1.97 (1.03–3.79)		0.04		0.79 (0.41–1.53		0.48		1.13 (0.76–1.69)		0.54
Eating sweets				< 0.001				0.03				0.78				0.31
Rarely or never		1.0 [Ref]				1.0 [Ref]				1.0 [Ref]				1.0 [Ref]		
Several times/wk		1.93 (1.43–2.59)		< 0.001		1.34 (0.82–2.18)		0.24		0.96 (0.71–1.32)		0.84		0.98 (0.51–1.91)		0.96
1 time/d		2.09 (1.54–2.83)		< 0.001		1.51 (0.91–2.49)		0.11		0.87 (0.62–1.24)		0.48		0.86 (0.51–1.44)		0.57
2–3 times/d		2.34 (1.79–3.32)		< 0.001		1.79 (1.06–3.03)		0.03		1.06 (0.74–1.50		0.76		0.82 (0.50–1.33)		0.42
≥ 4 times/d		2.22 (1.48–3.33)		< 0.001		1.01 (0.52–1.93)		0.99		0.99 (0.61–1.58)		0.96		0.88 (0.58–1.34)		0.56
Toothbrushing behavior																
Brushing frequency				0.01				0.77				0.01				0.51
Rarely or never		1.0 [Ref]				1.0 [Ref]				1.0 [Ref]				1.0 [Ref]		
Several times/wk		1.11 (0.89–1.40)		0.34		1.02 (0.50–2.10)		0.96		0.96 (0.71–1.31)		0.82		NA^a^		NA^a^
1 time/d		0.97 (0.78–1.21)		0.74		1.14 (0.62–2.10)		0.68		0.78 (0.57–1.05)		0.10		1.16 (0.73–1.85)		0.53
2–3 times/d		0.96 (0.77–1.21)		0.80		1.00 (0.55–1.83)		1.00		1.19 (0.88–1.60)		0.23		0.96 (0.70–1.31)		0.79
≥ 4 times/d		0.57 (0.40–0.82)		< 0.001		1.15 (0.63–2.13)		0.65		1.17 (0.78–1.75)		0.46		0.85 (0.63–1.15)		0.30
Brushing duration				0.01				0.21				< 0.001				< 0.001
< 1 min		1.0 [Ref]				1.0 [Ref]				1.0 [Ref]				1.0 [Ref]		
1 min		0.77 (0.63–0.94)		0.01		0.82 (0.58–1.17)		0.27		1.46 (1.10–1.93)		0.01		1.47 (1.05–2.05)		0.03
2 min		0.69 (0.55–0.86)		< 0.001		0.81 (0.61–1.08)		0.16		1.09 (0.81–1.47)		0.58		1.07 (0.75–1.52)		0.72
≥ 3 min		0.76 (0.59–0.99)		0.04		1.02 (0.78–1.34)		0.89		0.80 (0.52–1.21)		0.29		0.65 (0.39–1.07)		0.09
Dental visit history																
Past dental visit				< 0.001				< 0.001				< 0.001				< 0.001
Never		1.0 [Ref]				1.0 [Ref]				1.0 [Ref]				1.0 [Ref]		
Routine		1.02 (0.82–1.26)		0.87		1.04 (0.73–1.49)		0.83		1.63 (1.20–2.22)		< 0.001		1.45 (0.94–2.22)		0.09
Treatment		1.59 (1.29–1.96)		< 0.001		1.91 (1.44–2.52)		< 0.001		2.10 (1.69–2.62)		< 0.001		2.25 (1.70–2.99)		< 0.001
Emergency		1.96 (1.60–2.39)		< 0.001		2.01 (1.55–2.61)		< 0.001		2.13 (1.65–2.75)		< 0.001		2.61 (1.91–3.57)		< 0.001

*CI* confidence interval, *NA* not applicable, *RR* rate ratio

## Discussion

This cross-sectional study aimed to collect current data to assess the prevalence of dental caries and associated factors among first- and seventh-grade children in Tripoli, Libya. Findings indicated that more than three-quarters of first-grade and nearly one-half of seventh-grade children had dental caries, and caries severity among first-grade children was high. In addition, the majority of all children with caries had untreated decay, suggesting a lack of dental care visits to receive treatments such as fillings or extractions.

The total caries prevalence of 78.0% among first-grade children in our sample was consistent with the findings of Kumar et al. [3], which indicated that 78.6% of school-aged children living in Sebha, a large southern city in Libya, had dental caries; however, the caries prevalence among first-grade children in our study was substantially higher than the prevalence previously reported for the eastern and western areas of Libya. For example, 55% of children aged 6 years in Zawia and Zehra [10] and 63.5% of children in Benghazi [18] had tooth decay. Furthermore, the mean dmft of 3.7 among our sample of first-grade children in Tripoli was higher than that reported for children in any other city in Libya [10, 18].

Our study indicated that 48.2% of seventh-grade children in Tripoli had tooth decay in their permanent teeth, which was lower than the prevalence of tooth decay reported for children in Sebha [3] and Benghazi [19]. Our results were also lower than those of the only study to examine a similar population in Tripoli almost 3 decades ago, [12] which reported that 55.8% of fifth- and sixth-grade children had tooth decay in their permanent teeth, indicating a slight decrease in the rate of disease among the pediatric population. The DMFT scores among this age group (mean = 1.7 ± 1.6) were, however, similar to those reported for children in Benghazi within the same age group (mean = 1.7 ± 1.9). While our scores were much lower than the global target set by the World Health Organization for children in this age group (DMFT < 3.0), there is currently a call to use SiC scores to determine targets given the disproportionate distribution of caries among children [20].

Consistent with previous studies, parental employment status was significantly associated with the prevalence of untreated decay among seventh-grade children [21–23]. Children with mothers who were employed had less untreated decay and less severe caries than those with mothers who were homemakers. Mothers who were employed were likely to have high educational levels, which may be associated with increased knowledge of beneficial oral health–related behaviors. In addition, first- and seventh-grade children who were screened at the 2 centers in Hey Al-Andulus, which predominantly comprised children from high-income households, were less likely to have untreated decay. This finding may reflect the influence of socioeconomic status with regard to the oral health of children.

Our finding that the frequency of toothbrushing was not associated with decreases in tooth decay is inconsistent with results from several recent studies, which reported that more frequent toothbrushing was associated with lower DMFT, [8, 24, 25] reduced caries prevalence and risk, [24–26] and improvements in the dental health of young children [27]. However, brushing duration was associated with lower caries prevalence and less severe caries among seventh-grade children in our sample. Although the individual categories of toothbrushing duration were not statistically significant for first-grade children, toothbrushing duration overall was significantly associated with lower caries prevalence (*p* = 0.02), suggesting a cumulative effect of all categories. Nonetheless, fewer than one-third of the children brushed their teeth twice per day, and slightly more than one-third brushed their teeth for 2 min or more. While these results may be subject to bias associated with parental reporting, they nevertheless indicate that the brushing habits of the majority of children did not meet professional recommendations, which encourage brushing twice per day for 2 min [28]. These findings highlight the need for health education programs focusing on behavior change to improve brushing habits.

In the present study, the prevalence and severity of untreated decay were lower among first-grade children who consumed less sugar, which aligns with studies reporting that (1) consumption of sugar is associated with caries; (2) children who frequently consume sugar, particularly before bedtime, are more likely to develop caries; and (3) intake of dietary sugars is the most important risk factor for the development of caries [29–32]. Interestingly, sugar intake from all sources was not significantly associated with dental caries among the seventh-grade children in our sample. This finding differs from the results of a previous study of children aged 12 years in Benghazi, which reported that the consumption of fruit-based juice and power drinks was associated with dental caries [33].

In general, low sugar intake was reported among the seventh-grade children in our study. For example, the majority of seventh-grade children (80.1%, n = 714) consumed sweets once per day or less, as reported by parents. However, the prevalence of untreated decay was generally high in our sample, even among children whose parents rarely reported consumption of the 3 sources of sugar included in the survey. This result may indicate that our survey did not capture all sources of sugar, such as added sugar, power drinks, or flavored milk, or that it did not account for sources of fluoride in drinking water. Given that the survey relied on parental reporting, this finding may also indicate that the responses for brushing frequency and duration were subject to bias, as the survey did not ask parents how they controlled and monitored their child’s brushing frequency and duration. We also did not control for the possibility that parents may have provided socially desirable, but inaccurate, responses regarding sugar consumption, brushing frequency, and brushing duration.

Our finding that children who had never visited the dentist had less untreated decay than those who had visited the dentist (either for treatment or emergencies) is suggestive of a common culture of disease-oriented care-seeking behaviors among families in Tripoli. This finding also aligns with the results of previous studies from developing countries, which concluded that the high prevalence of dental caries was associated with dental visit history [34, 35].

Nearly two-thirds of parents in our study reported that lack of pain or lack of dental need were primary barriers to dental care utilization, and routine dental visits within the past year were reported for barely one-third of the children. These results are concerning, as the American Academy of Pediatric Dentistry recommends that children visit the dentist every 6 months beginning at age 12 months, and dental visits scheduled for preventive reasons are strong predictors of oral health [36, 37].

This study has several limitations, primary of which was the lack of randomization due to the use of convenience sampling, which is susceptible to bias and selection errors [13]. However, this limitation was partly mitigated by the fact that the school-entry examination was mandatory for all children who presented at the screening sites. In addition, we oversampled to diminish the possibility of sampling errors [13]. Thus, although the sample was not randomly selected, it represented approximately 10% of the entire population of first- and seventh-grade children in Tripoli. We also assumed that children would receive their school-entry examinations at health centers located in their home districts; however, they were permitted to receive screenings at any participating center, which might have resulted in underestimation of the differences between the districts.

Household income data were not collected due to the sensitivity of the topic, as questions about income are considered culturally inappropriate in Libya. Therefore, school type and screening site were used as proxies for family income. Likewise, and in contrast to Western countries, census data about race and ethnicity are not routinely collected or referenced. For many years, the country functioned under the normative assumption that all Libyan citizens were of Arab descent, and ethnic and linguistic diversity was not tolerated; this intolerance resulted in the marginalization of minority populations, including those of African, Berber, Tuareg, and Tabu ancestry [38, 39]. Raising awareness of the importance of collecting socioeconomic and demographic data may help to reveal disparities in health outcomes among minority groups.

The study may also have been subject to social desirability bias due to the potential overreporting of desirable oral health–related behaviors, such as frequent toothbrushing and low sugar consumption. Future studies should consider the use of social desirability measures to control for this type of bias. In addition, our analysis used cross-sectional data, from which it was not possible to measure causality. Follow-up surveys are warranted to estimate the true prevalence of disease. Finally, agreement between examiners (via calculation of κ statistics or other values) was not measured; future research should incorporate such measures to determine fidelity of dental examiners’ assessments of children’s teeth, both within and between screening sites.

Our findings indicated that the majority of first-grade children and almost one-half of seventh-grade children had untreated dental caries, which suggests that continued efforts are needed to improve oral health care utilization among school-aged children in Tripoli, Libya. We also found that screening site, maternal employment status (among seventh-grade children), brushing duration (among seventh-grade children), past dental treatment, and past emergency visit were the most significant factors associated with caries prevalence. The influence of socioeconomic status on children’s oral health that was observed in this study provides reason for concern, as it suggests that discrepancies may be widespread across socioeconomic groups in the community. This issue is a critical one in Libya, which faces other urgent public health problems fueled by the aftermath of the Arab Spring. These problems include violence leading to injuries and death, population displacement, increasing unemployment rates and food costs, and the need for public assistance programs [40]. Addressing these tremendous oral and general public health problems will require collaboration within and between all sectors and will necessitate the building of local infrastructures and increased capacity for public health programs that focus on health promotion and disease prevention.

## Conclusion

In this study, a high prevalence of dental caries was found among first- and seventh-grade children in Tripoli, Libya. This high prevalence was significantly associated with socioeconomic factors, such as screening site and maternal employment, as well as behavioral factors, such as toothbrushing duration, past dental treatment, and past emergency visit. These findings can be used to evaluate current public health initiatives and inform future program and policy planning. The results may also provide a framework for ongoing and future public health surveillance efforts in Tripoli.

## Supplementary Information

**Additional file 1**: Parental Survey About Children’s Oral Habits, Tripoli, Libya. Copy of the parental survey used in the study.

## Data Availability

The data that support the findings of this study are available from the Tripoli Bureau of Primary Care, but restrictions apply to the availability of these data, which were used under license for the current study, and so are not publicly available. Data are, however, available from the authors upon reasonable request and with permission of the Tripoli Bureau of Primary Care.

## Abbreviations

dmft: Decayed, missing, or filled teeth (primary teeth)
DMFT: Decayed, missing, or filled teeth (permanent teeth
