# Supplementary material for: Prevalence of dental caries and associated factors among school-aged children in Tripoli, Libya: a cross-sectional study
Source: BMC Oral Health. 2021 Apr 30;21:224. doi: 10.1186/s12903-021-01545-9 (PMC8086357; doi:10.1186/s12903-021-01545-9)
Supplement: Supplementary file 1 — Additional file 1: Parental Survey About Children’s Oral Habits, Tripoli, Libya. Copy of the parental survey used in the study. [file 12903_2021_1545_MOESM1_ESM.docx]

**إستبيان**

**ا-معلومات إجتماعية وديموغرافية**

1. ماهو جنس طفلك؟ ذكر أنثي
2. في أي صف دراسي سوف يدرس طفلك؟

الصف الأول الصف الأول أعدادي

1. ماهو نوع مدرسة طفلك؟

مدرسة خاصة مدرسة عامة

5. وظيفة الأب.........................

**ب-ممارسات متعلقة بالأسنان**

1) خلال الإسبوع الماضي, في العادة كم مرة في اليوم الواحد قام طفلك بشرب المشروبات الغازية مثل البيبسي أو الكوكا كولا او السفن آب أوغيرها؟

1. لم يشرب طفلي أي مشروبات غازية في الإسبوع الماضي
2. عدة مرات في الإسبوع ولكن ليس كل يوم
3. مرة واحدة في اليوم
4. مرتان أو ثلاث مرات في اليوم
5. 4مرات أوأكثر
6. لاأعلم

2) خلال الإسبوع الماضي, في العادة كم مرة قام طفلك بشرب العصائرغيرالطبيعية مثل عصير الريحان أو السن توب وغيرها؟

1. لم يشرب طفلي أي عصائرغير طبيعية خلال الإسبوع الماضي
2. عدة مرات في الإسبوع ولكن ليس كل يوم
3. مرة واحدة في اليوم
4. مرتان أو ثلاث مرات في اليوم
5. 4 مرات في اليوم أوأكثر
6. لا أعلم

3)خلال الإسبوع الماضي, عادة كم مرة في اليوم الواحد قام طفلك بتنظيف أسنانه بالفرشاة والمعجون ؟

1. لم يقم طفلي مطلقا بتنظيف أسنانه خلال الإسبوع الماضي
2. عدة مرات في الإسبوع, و ليس كل يوم
3. مرة واحدة يوميا
4. مرتين يوميا
5. 3 مرات يوميا أو أكثر
6. لاأعلم

4) خلال الإسبوع الماضي, في العادة كم مرة في اليوم الواحد قام طفلك بأكل الحلويات مثل الكيك, والشكولاطة, والحلوي وغيرها ؟

1. لم يقم طفلي بأكل الحلويات خلال الإسبوع الماضي

وظيفة الأم........................

1. المستوي التعليمي للأب:

متحصل علي شهادة ثانوية أوأعلي

غير متحصل شهادة ثانوية

1. المستوي التعليمي للأم:

متحصلة علي شهاد ثانوية أوأعلي

غير متحصلة علي شهادة ثانوية

___________________________

1. مرة واحدة في اليوم
2. مرتين أو ثلاث مرات في اليوم
3. 4مرات أوأكثر

5)ماهي المدة التي يقضيها طفلك في تنظيف أسنانه؟

1. أقل من نصف دقيقة
2. لمدة دقيقة واحدة
3. لمدة دقيقتين
4. لمدة 3 دقائق أو أكثر
5. لاأعلم

6)متي كانت أخر مرة زار فيها طفلك طبيب الأسنان؟ (عدا الزيارات الخاصة بالمدارس)

1. خلال السنة الماضية
2. قبل أكثر من سنة
3. لم يزر طبيب الإسنان مطلقا
4. لاأعلم

7)ما السبب الرئيسي لزيارة طفلك طبيب الأسنان في أخر مرة؟

1. لغرض الكشف الدوري لفحص الأسنان أوتنظيفها
2. للقيام بعلاج مثل الحشو أو الخلع وغيره
3. زيارة مستعجلة نتيجة ألام الأسنان أو التهاب الفم وانتفاخه
4. طفلي لم يزر طبيب الأسنان اطلاقا
5. لاأعلم

8) ماهو السبب الرئيسي الذي يمنعك من اصطحاب طفلك لزيارة طبيب الأسنان ؟

1. عدم القدرة المادية
2. عدم توفر الوقت
3. **
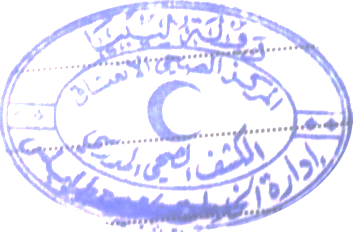
**عدم المعرفة إلي أين أذهب
4. عدم الثقة بأطباء الأسنان
5. أسباب أخري..أذكرها....
